# Outcomes for Elderly Patients Aged 70 to 80 Years or Older with Locally Advanced Oral Cavity Squamous Cell Carcinoma: A Propensity Score–Matched, Nationwide, Oldest Old Patient–Based Cohort Study

**DOI:** 10.3390/cancers12020258

**Published:** 2020-01-21

**Authors:** Ben-Chang Shia, Lei Qin, Kuan-Chou Lin, Chih-Yuan Fang, Lo-Lin Tsai, Yi-Wei Kao, Szu-Yuan Wu

**Affiliations:** 1 Research Center of Big Data, College of management, Taipei Medical University, Taipei 110, Taiwan; stat1001@tmu.edu.tw; 2 College of Management, Taipei Medical University, Taipei 110, Taiwan; 3 Executive Master Program of Business Administration in Biotechnology, College of Management, Taipei Medical University, Taipei 110, Taiwan; 4 School of Statistics, University of International Business and Economics, Beijing 100029, China; qinlei@uibe.edu.cn; 5 Division of Oral and Maxillofacial surgery, Department of Dentistry, Wan Fang Hospital, Taipei Medical University, Taipei 110, Taiwan; kclin0628@hotmail.com (K.-C.L.); ndmcd52@gmail.com (C.-Y.F.); lolintsai@tmu.edu.tw (L.-L.T.); 6 School of Dentistry, College of Oral Medicine, Taipei Medical University, Taipei 110, Taiwan; 7 Graduate Institute of Business Administration, Fu Jen Catholic University, Taipei 242, Taiwan; kyw498762030@gmail.com; 8 Department of Food Nutrition and Health Biotechnology, College of Medical and Health Science, Asia University, Taichung 41354, Taiwan; 9 Division of Radiation Oncology, Lo-Hsu Medical Foundation, Lotung Poh-Ai Hospital, Yilan 265, Taiwan; 10 Big Data Center, Lo-Hsu Medical Foundation, Lotung Poh-Ai Hospital, Yilan 265, Taiwan; 11 Department of Healthcare Administration, College of Medical and Health Science, Asia University, Taichung 41354, Taiwan; 12 Department of Radiology, School of Medicine, College of Medicine, Taipei Medical University, Taipei 110, Taiwan

**Keywords:** old, oral cavity squamous cell carcinoma, locally advanced, treatments, mortality

## Abstract

*Purpose*: Although clinicians encounter patients aged ≥70 years with locally advanced oral cavity squamous cell carcinoma (LA-OCSCC), no evidence is available to facilitate decision making regarding treatment for this elderly population. *Methods*: We selected elderly (≥70 years) patients from the Taiwan Cancer Registry database who had received a diagnosis of LA-OCSCC. Propensity score matching was performed. Cox proportional hazards model curves were used to analyze all-cause mortality in patients in different age groups receiving different treatments. *Results*: The matching process yielded a final cohort of 976 patients in concurrent chemoradiotherapy (CCRT), non-treatment, radiotherapy (RT) alone, and surgery cohorts who were eligible for further analysis. After stratified analysis, the adjusted hazard ratios (aHRs) (95% confidence intervals [CIs]) derived for surgery, RT alone, and non-treatment compared with CCRT were 0.66 (0.52 to 0.83), 1.02 (0.81 to 1.28), and 1.52 (1.21 to 1.91), respectively, in patients aged 70 to 80 years. In the oldest patients (aged >80 years), multivariate analysis indicated that the results of surgery or RT alone were nonsignificant compared with those of CCRT. The aHR (95% CI) derived for the highest mortality was 1.81 (1.11 to 2.40) for non-treatment compared with CCRT. *Conclusions*: Surgery for elderly patients with LA-OCSCC is associated with a significant survival benefit, but the association is nonsignificant in the oldest elderly patients. No survival differences were observed between RT alone and CCRT in these elderly patients. Non-treatment should not be an option for these patients.

## 1. Introduction

Head and neck squamous cell carcinoma (HNSCC) is endemic in Asia, particularly in Taiwan and India [1,2,3]. Betel nut is a distinctive carcinogen consumed in Taiwan and causes oral cavity, oropharyngeal, hypopharyngeal, and laryngeal cancers [1,2,3,4,5,6,7,8] In Taiwan, more than 80% of HNSCCs originate in the oral cavity rather than the oropharynx [1,2,3,4,5,6,7,8]. The distribution of HNSCCs in Taiwan differs from that in other countries, where most HNSCCs are oropharyngeal squamous cell carcinomas [9,10,11]. Oral cavity squamous cell carcinoma (OCSCC) caused by chewing betel nut has more frequent locoregional recurrence and less distant metastasis [1,2,3,4]. Local treatments including surgery, radiotherapy (RT), or concurrent chemoradiotherapy (CCRT) are crucial in reducing locoregional failure and improving overall survival (OS) in patients with OCSCC [1,2,3,4,5,6,7,8].

According to National Comprehensive Cancer Network (NCCN) guidelines, surgery is the main treatment for resectable OCSCC in the United States [12]. Taiwanese cancer physicians typically follow the NCCN guidelines because the Taiwan National Health Research Institute Clinical Cancer Quality Certification Program regularly assesses the rate of compliance with these cancer guidelines [13]. Physicians not complying with the cancer treatment guidelines do not pass quality certification and are penalized by the Health Promotion Administration of the Taiwan Ministry of Health and Welfare [13]. However, elderly patients (aged >70 years) with OCSCC are scarcely mentioned in clinical trials and cancer treatment guidelines [12,14]. Old age remains a critical problem in therapeutic decision making for patients with OCSCC receiving surgery, RT alone, CCRT, or even non-treatment [11,15,16]. Taiwanese patients with OCSCC tend to be the main economic provider in their family and are younger (mean age: 53 years) than those in Western countries (mean age: 62 years) [3,4,5,7,8,13,17,18]. The medical institutions in Taiwan thus have less experience in treating patients 70 years or older with OCSCC than those in other countries. Higher mortality in surgery or CCRT was reported in elderly patients with HNSCC, particularly those older than 70 years [11,16]. Adding chemotherapy to RT in patients older than 80 years with HNSCC might be too toxic and thus reduce survival [11]. The definition of “old age” in patients with locally advanced OCSCC (LA-OCSCC) who cannot tolerate aggressive treatment or even non-treatment after comorbidity control remains unclear, and the optimal treatment for these “old” patients with LA-OCSCC remains undetermined.

In contrast to oropharyngeal cancer, OCSCC is endemic in Taiwan [1,2,3,4,5,6,7,8,16]. A previous study could not determine the optimal treatment for elderly patients with LA-OCSCC [11]. Although clinicians encounter patients 70 years or older with LA-OCSCC, no evidence is currently available that can inform treatment decision making for this population. The present study evaluated practice patterns and outcomes associated with treatment strategies for this elderly population. Furthermore, we performed a propensity score matching (PSM) study with a nationwide cohort by using the Taiwan Cancer Registry Database (TCRD) to determine the optimal treatment for elderly patients with LA-OCSCC and estimate the survival effect of different treatments in these patients.

## 2. Patients and Methods

We selected elderly patients (aged ≥70 years) from the TCRD who had received a diagnosis of OCSCC between 1 January 2006, and 31 December 2015. The follow-up period was from the index date to 31 December 2016. To address immortal time bias, we modified the index date of the cohorts. The index date was the date of surgery in the surgery cohort, the start date for RT in the CCRT and RT-alone cohorts, and the date of OCSCC diagnosis in the non-treatment cohort. Elderly patients who received treatment for more than 3 months after receiving a diagnosis of OCSCC were excluded to prevent the immortal period in the treatment cohorts when compared with the non-treatment cohort. The survival benefits were underestimated in the treatment cohorts compared with the non-treatment cohort. The advantages of treatments were underestimated only when compared with the non-treatment cohort. After adjustment for confounders, a time-dependent Cox proportional hazards model was constructed to model the time from the index date to all-cause mortality in elderly patients receiving treatment. Our protocols were reviewed and approved by the Institutional Review Board of Taipei Medical University (TMU-JIRB 201702019). The Collaboration Center of Health Information Application TCRD contains detailed cancer-related information regarding the clinical stage and the RT dose, RT technique, and chemotherapy regimen used [19,20,21,22,23,24,25,26,27,28]. The diagnoses of selected patients were confirmed on the basis of pathological data. Furthermore, we confirmed that patients who had received a new diagnosis of OCSCC had no other cancers or distant metastasis. The inclusion criteria were OCSCC diagnosis; age ≥70 years; and American Joint Committee on Cancer (AJCC) Cancer Staging Manual, Seventh Edition clinical cancer stages III and IV (without distant metastasis). The exclusion criteria were history of cancer before OCSCC diagnosis, distant metastasis, missing sex data, unclear staging, and non–squamous cell carcinoma histology. OCSCC was defined as squamous cell carcinoma within the oral cavity with pathological confirmation recorded in the TCRD. In addition, we excluded patients with unresectable OCSCC who received RT alone or CCRT after receiving the diagnosis, those who did not receive cisplatin-based chemotherapy regimens, and those who received therapy for more than 12 weeks after receiving the diagnosis. Finally, we selected patients with LA-OCSCC, regardless of whether they received therapy, and categorized them into the following groups on the basis of treatment modality for outcome comparison: group 1, those receiving definitive CCRT; group 2, those not receiving treatment; group 3, those receiving RT alone; and group 4, those receiving surgery. Adjuvant therapies were included in group 4. The median total dose and fraction size of RT were 70 and 2 cGy, respectively, in groups 1 and 3. Comorbidities were scored using the Charlson Comorbidity Index (CCI) [8,29]. Our CCI scores did not include history of cancer before OCSCC diagnosis, age, or distant metastasis in our cohort on the basis of the exclusion criteria to prevent duplicate weighting calculations for survival effects. Only comorbidities observed 6 months before the index date were included. Comorbid conditions were identified and included according to the International Classification of Diseases, Ninth Revision, Clinical Modification (ICD-9-CM) diagnostic codes for the first admission or more than two repeated main diagnosis codes for visits to the outpatient department. Data Availability: We used data from the TCRD and National Health Insurance Research Database (NHIRD). The authors confirm that, for approved reasons, some access restrictions apply to the data underlying the findings. The data utilized in this study cannot be made available in the manuscript, the supplemental files, or in a public repository due to the “Personal Information Protection Act” executed by Taiwan’s government, starting from 2012. Requests for data can be sent as a formal proposal to obtain approval from the ethics review committee of the appropriate governmental department in Taiwan. Specifically, links regarding contact info for which data requests may be sent to are as follows: http://nhird.nhri.org.tw/en/Data_Subsets.html#S3 and http://nhis.nhri.org.tw/point.html. Owing to aforementioned reasons, we have approval of IRB and patients consents were not necessary.

To reduce the effects of potential confounding factors when comparing therapy outcomes between the groups, a propensity score (PS) was estimated using a multivariate logistic regression model with the treatment group as the dependent variable and potential confounders as covariates. Three logistic regressions were used for PSM, and patients receiving CCRT were used as the reference group. Interpretation and use of the Cox proportional hazards model was dependent on the proportional hazards assumption. Thus, our model assumptions were proportional to the hazards between the different treatments and case-mix variables based on the study by Grambsch [30], who proposed a practical test and an associated graph for examining the critical assumption. A significant *p* value for the global test indicates that the proportional hazards assumption is violated for the covariate [30]. Because Schoenfeld residuals are based on the assumption that the effects of predictor variables are independent of time, a plot of Schoenfeld residuals versus time can be evaluated to determine whether the effect of the predictor variable changes during the follow-up period. Both the global test and plot assessment indicated that the proportional hazard assumptions were true for the data set in this study. The proportionality of the hazards between the treatment and case-mix variables was assessed. Specifically, we first obtained PSs by using logistic regression on receiving different treatments for the variables age; sex; medical center; betel nut use; cigarette smoking; alcohol consumption; AJCC clinical stage; tumor, node, and metastasis (TNM) stage; and CCI. The second and third comparable control groups were iteratively matched on the basis of the PSs calculated from the two other logistic regressions. All patients in group 2 were matched at a 1:1 ratio with patients in groups 1, 3, and 4 through PSM by using the global optimization method [31]. Multivariate Cox regression analysis was performed to calculate the hazard ratio (HR) for determining whether factors such as therapy type, age, sex, CCI, medical center, betel nut use, cigarette smoking, alcohol consumption, AJCC clinical stage, TNM stage, and CCI were significant independent predictors. The independent predictors were controlled in the analysis, and the endpoint was all-cause mortality in the treatment groups, with group 1 (CCRT) serving as the control arm.

All-cause mortality was estimated using time-dependent Cox proportional hazard curves for OS in patients receiving different treatments and in different age intervals. After adjustment for confounders, the time-dependent Cox proportional hazards method was used to model the time from the index date to all-cause mortality in patients receiving treatment. In the multivariate analysis, HRs were adjusted for age, sex, medical center, CCI, betel nut use, cigarette smoking, alcohol consumption, AJCC clinical stage, and TNM stage. All analyses were performed using SAS version 9.3 (SAS Institute, Cary, NC, USA). A 2-tailed *p* < 0.05 was considered significant. Overall and age-specific (patients aged 70–80 and ≥81 years) survival rates were calculated.

## 3. Results

The matching process yielded a final cohort of 976 elderly patients (256, 227, 237 and 256 patients in groups 1, 2, 3, and 4, respectively) who were eligible for further analysis; their characteristics are summarized in Table 1. The age distribution by 10-year age intervals was balanced among the 4 groups (Table 1). AJCC clinical stages were identical among the treatment groups. In the 4 cohorts, 20.7% and 79.3% of the patients had disease in AJCC clinical stage III and IV, respectively. Moreover, CCI scores, medical centers, and betel nut use, cigarette smoking, and alcohol consumption habits were similar among the four cohorts. Follow-up duration was not matched in the analysis because survival time was inconsistent among the treatment groups (Table 1). Approximately 50% of patients had clinical T4 and N2 stages.

According to the multivariate Cox regression analysis, treatment was a significant predictor of all-cause mortality (Table 2). Both univariate and multivariate Cox regression analyses indicated that surgery was associated with the highest OS. However, CCRT outcomes were not significantly different from those of RT alone. The HR for the univariate model was similar to that in the multivariate Cox regression analyses. The type III assessments of the significance (*p* values) of each explanatory variable (with more than two levels) are presented in Table 2, Table 3 and Table 4. No significant differences were observed in the explanatory variables except for treatment difference and clinical stage (Table 2). In multivariate Cox regression analyses, the adjusted HRs (aHRs) (95% confidence intervals [CIs]) for surgery, RT alone, and nontreatment compared with CCRT were 0.69 (0.55 to 0.85), 1.06 (0.87 to 1.31), and 1.60 (1.30 to 1.97), respectively.

After stratified analysis of old (70–80 years) and the oldest (≥81 years) patients with LA-OCSCC, multivariate Cox regression analysis revealed that surgery in elderly patients 70 to 80 years old was associated with the highest OS compared with other treatments. The aHRs for surgery, RT alone, and non-treatment compared with CCRT were 0.66 (0.52 to 0.83), 1.02 (0.81 to 1.28), and 1.52 (1.21 to 1.91), respectively, in elderly patients with LA-OCSCC (Table 3). Survival remained significantly the lowest in the non-treatment cohort (Table 3). In the oldest (≥81 years) patients with LA-OCSCC, univariate and multivariate Cox regression analyses indicated that surgery or RT alone did not significantly affect OS compared with the CCRT (Table 4). The aHR (95% CI) derived for significant independent prognostic risk factors for poor OS was 1.81 (1.11 to 2.40) for non-treatment compared with CCRT (Table 4).

Figures S1–S3 present the predicted Cox proportional hazard curves for OS obtained for elderly patients receiving different treatments after adjustment for age, sex, medical center, CCI, betel nut use, cigarette smoking, alcohol consumption, AJCC clinical stage, and TNM stage. The most improved OS curve was obtained for surgery, followed by RT alone and CCRT (both equally improved) and then non-treatment in elderly patients with LA-OCSCC (Figures S1–S3).

## 4. Discussion

Surgery is generally recommended as the initial therapy for LA-OCSCC [12]. RT alone and CCRT are alternatives for patients who refuse surgery or have unresectable LA-OCSCC, but these treatments are not recommended for resectable LA-OCSCC [3,4,12]. Currently, data from randomized clinical trials (RCTs) attempting to define an optimal strategy for patients with stages III and IV OSCC, particularly elderly patients, are inadequate. Moreover, elderly patients with LA-OSCC have rarely been enrolled in RCTs because older adults, particularly those older than 70 years, are often underrepresented in clinical trials [14]. Therefore, no head-to-head RCT or large cohort study has estimated the survival effects of different treatment strategies in elderly patients with LA-OSCC. The meta-analysis of chemotherapy in head and neck cancer (MACH-NC) revealed that older patients were less likely to benefit from chemotherapy additional to definitive locoregional treatment, and the meta-analysis could not demonstrate a benefit in patients older than 70 years [11]. In fact, the meta-analysis suggested that additional chemotherapy may be harmful for patients older than 80 years [11]. CCRT is often avoided in older patients with advanced oropharyngeal cancer as well as those with multiple comorbidities because it may delay or prevent completion of a definitive RT course [11]. In addition, only 21.34% of patients with OCSCC in clinical stages I through IV were included in the MACH-NC meta-analysis [11]. Currently, no definitive evidence supports using surgery, RT alone, non-treatment, or CCRT in elderly patients with LA-OCSCC.

Table 1 indicates that PSM was performed efficiently. Most covariates of OS in OCSCC were balanced among different treatments in elderly patients with LA-OCSCC. Age, sex, CCI, medical center, AJCC clinical stage, TNM stage, alcohol consumption, betel nut use, and cigarette smoking were equal in the 4 cohorts. Old age, male sex, high CCI score, nonmedical center, AJCC clinical stage, TNM stage, alcohol consumption, betel nut use, and cigarette smoking are poor prognostic factors for OS in patients with OCSCC [3,4,5,6,7,8,11,15,16]. After PSM, we could estimate the survival outcomes of different treatments in elderly patients with LA-OCSCC within balanced matching conditions [32].

Our findings revealed no statistical significance between definitive RT alone and definitive CCRT in elderly (≥70 years) patients with LA-OCSCC (Table 2 and Figure S2). These findings are similar to those of the MACH-NC, in which adding chemotherapy to RT was not beneficial to OS in elderly patients with HNSCC [11]. However, the patients in our population had only LA-OCSCC, whereas most of the patients in the MACH-NC had oropharyngeal cancer [11]. Therefore, the present study is the first study to compare RT alone with CCRT in elderly patients with LA-OCSCC. Our findings suggest that CCRT is not associated with better survival than RT alone after controlling for covariates of age, sex, comorbidities, medical center with larger patient volume, clinical stage, alcohol consumption, betel nut use, and cigarette smoking. Moreover, surgery in elderly patients with LA-OCSCC was discovered to be valuable and resulted in lower all-cause mortality than definitive CCRT; this may have been because elderly patients with LA-OCSCC may live to experience disease progression after receiving CCRT instead of surgery [33]. However, evidence of the benefits of surgery in the oldest (>80 years) patients with LA-OCSCC remains elusive, and this treatment should be investigated prospectively. Our study is also the first to demonstrate that surgery remains the optimal therapy for elderly patients with LA-OCSCC rather than RT alone or CCRT. Elderly patients with LA-OCSCC must continue to receive surgery as the first option in cancer treatment, as stated in the NCCN guidelines [12]. If elderly patients with LA-OSCC refuse surgery, RT alone is sufficient, and CCRT might be unnecessary. Non-treatment in elderly patients with LA-OCSCC resulted in the lowest survival rate (Table 2).

According to the MACH-NC, additional chemotherapy in patients older than 80 years with HNSCC may be harmful [11]. Therefore, we performed stratified analysis of elderly (70–80 years, Table 3) and the oldest (>80 years, Table 4) patients with LA-OCSCC to estimate the survival effects of various treatments in these age groups. The survival effects of different treatments in elderly patients with LA-OCSCC were similar to the results presented in Table 2. RT alone in elderly patients with LA-OCSCC was sufficient but nonsignificant compared with definitive CCRT. Surgery was again discovered to be the optimal therapy rather than CCRT or RT alone. Non-treatment had the lowest survival rate after PSM. Marginal effects remained clear in clinical stages, as presented in Table 2 [34]. For the oldest (>80 years) patients with LA-OCSCC, CCRT was not harmful compared with RT alone, and its effect was inconsistent with the results for patients with HNSCC in the MACH-NC. Notably, definitive CCRT was superior to RT alone, but the difference was nonsignificant. A possible reason for this is that the MACH-NC included more elderly patients with oropharyngeal cancer who responded well to RT alone and benefited less from chemotherapy with RT, particularly in patients with human papillomavirus-driven oropharyngeal cancer [11,35,36]. Moreover, the effect of surgery was nonsignificant in terms of survival benefit compared with definitive CCRT (Table 4). In the oldest patients with LA-OCSCC, surgical mortality was higher, which masked the survival benefits [15,16]. However, the survival rate for non-treatment remained the lowest. Although these findings suggest no significant survival differences among RT alone, CCRT, and surgery in the oldest patients with LA-OCSCC after PSM, receiving a single treatment was more effective than non-treatment for the oldest patients with LA-OCSCC.

The strengths of this study are its large sample size and homogenous LA-OCSCC population with old and oldest age cohorts. The population in this study had homogeneous cancer sites and pathologies (all OCSCC), similar clinical stages, and homogeneous RT doses. Most major covariates—age, sex, CCI, medical center, AJCC clinical stage, TNM stage, alcohol consumption, betel nut use, and cigarette smoking—were considered in PSM analysis. To our knowledge, this is the first and largest PSM study to estimate the effects of surgery, RT alone, and non-treatment compared with definitive CCRT. In elderly patients with LA-OCSCC (aged 70–80 years), surgery was more beneficial than definitive CCRT, and a nonsignificant difference was observed between RT alone and CCRT (Table 3). No survival differences were observed among surgery, CCRT, and RT alone in the oldest (aged >80 years) patients with LA-OCSCC (Table 4). Non-treatment in elderly patients with LA-OCSCC resulted in the lowest survival rate regardless of age cohort. These findings should be considered in future clinical practice and prospective clinical trials.

This study had some limitations. First, because all elderly patients with LA-OCSCC were enrolled from an Asian population in an area with prevalent betel nut use, the corresponding ethnic susceptibility remains unclear. Hence, our results should be cautiously extrapolated to non-Asian populations in areas where betel nut use is not prevalent. Second, diagnoses of all comorbid conditions were based on ICD-9-CM codes. Nevertheless, the Taiwan Cancer Registry Administration randomly reviews charts and interviews patients to verify diagnostic accuracy, and hospitals with outlier charges or practices may be audited and subsequently penalized heavily if malpractice or discrepancies are identified. Therefore, to obtain crucial information on population specificity and disease occurrence, a large-scale randomized trial comparing carefully selected patients receiving suitable treatments is essential. Third, the sample size of the oldest patients with LA-OCSCC was 169 after PSM. However, a narrower CI implies a smaller chance of obtaining an observation within that interval. Therefore, our accuracy was higher (Table 4) [37,38]. Fourth, the sample size was insufficient for performing additional PSM to estimate the 2 age groups (70–80 and >80 years), particularly in the oldest patients (≥81 years) with LA-OCSCC. Bias may have existed in the variables of age, sex, and medical center when the patient group was divided. However, multivariate analyses were performed using the Cox proportional hazards model for mortality risk between the 2 groups of elderly patients with LA-OCSCC (Table 3 and Table 4). Finally, the TCRD does not contain information regarding dietary habits, socioeconomic status, or body mass index, which may be risk factors for mortality. However, because of the magnitude and statistical significance of the observed effects in this study, these limitations were unlikely to have affected the conclusions.

## 5. Conclusions

For elderly (aged 70–80 years) patients with LA-OCSCC, surgery is the first option for treatment, and no survival difference between RT alone and CCRT was discovered. In the oldest (aged >80 years) patients with LA-OCSCC, no survival differences were observed among surgery, RT alone, and CCRT. Non-treatment in both age groups resulted in the lowest survival rate. Therefore, to obtain crucial information on population specificity and disease occurrence, a large-scale randomized trial comparing carefully selected patients receiving suitable treatment is essential.

## Figures and Tables

**Table 1:** Characteristics of Elderly Patients (≥70 years) with Locally Advanced Oral Cavity Squamous Cell Carcinoma who Received Different Treatments and Propensity-Score-Matched Cohorts.

Variable		Total, *n* (%) (*n* = 976)	CCRT, *n* (%) (*n* = 256)	Nontreatment, *n* (%) (*n* = 227)	RT Alone, *n* (%) (*n* = 237)	Surgery, *n* (%) (*n* = 256)	*p* Value
Sex							0.536
Female		159 (16.3)	36 (14.1)	38 (16.7)	37 (15.6)	48 (18.8)	
Male		817 (83.7)	220 (85.9)	189 (83.3)	200 (84.4)	208 (81.2)	
Age (y)							0.876
70 to 80		807 (82.7)	213 (83.2)	185 (81.5)	194 (81.9)	215 (84.0)	
≥81		169 (17.3)	43 (16.8)	42 (18.5)	43 (18.1)	41 (16.0)	
CCI score							0.962
0		715 (73.3)	187 (73.0)	170 (74.9)	173 (73.0)	185 (72.3)	
1		35 (3.6)	9 (3.5)	7 (3.1)	9 (3.8)	10 (3.9)	
2		123 (12.6)	31 (12.1)	25 (11.0)	35 (14.8)	32 (12.5)	
3		34 (3.5)	8 (3.1)	10 (4.4)	8 (3.4)	8 (3.1)	
4+		69 (7.1)	21 (8.2)	15 (6.6)	12 (5.1)	21 (8.2)	
Mean (SD)		0.86 (1.90)	0.92 (2.02)	0.79 (1.72)	0.76 (1.62)	0.97 (2.15)	0.536
AJCC clinical stage							0.447
III		202 (20.7)	46 (18.0)	44 (19.4)	53 (22.4)	59 (23.0)	
IV		774 (79.3)	210 (82.0)	183 (80.6)	184 (77.6)	197 (77.0)	
TNM stage							
T stage							0.583
T1		49 (5.0)	13 (5.1)	11 (4.8)	12 (5.1)	13 (5.1)	
T2		199 (20.4)	59 (23.0)	42 (18.5)	42 (17.7)	56 (21.9)	
T3		181 (18.5)	42 (16.4)	38 (16.7)	44 (18.6)	57 (22.3)	
T4		547 (56.0)	142 (55.5)	136 (59.9)	139 (58.6)	130 (50.8)	
N stage							0.769
N0	0	218 (22.3)	52 (20.3)	57 (25.1)	56 (23.6)	53 (20.7)	
N1	1	227 (23.3)	60 (23.4)	44 (19.4)	62 (26.2)	61 (23.8)	
N2	2	495 (50.7)	134 (52.3)	116 (51.1)	111 (46.8)	134 (52.3)	
N3	3	36 (3.7)	10 (3.9)	10 (4.4)	8 (3.4)	8 (3.1)	
M stage	0	976 (100.0)	256 (100.0)	227 (100.0)	237 (100.0)	256 (100.0)	1.000
Medical center							0.882
Medical center		289 (29.6)	33 (29.7)	27 (29.5)	28 (29.5)	26 (29.7)	
Nonmedical center		687 (70.4)	180 (70.3)	160 (70.5)	167 (70.5)	180 (70.3)	
Cigarette smoking							0.871
No		290 (29.7)	75 (29.3)	68 (30.0)	71 (30.0)	76 (29.7)	
Yes		686 (70.3)	181 (70.7)	159 (70.0)	166 (70.0)	180 (70.3)	
Betel nut use							0.691
No		118 (12.1)	31 (12.1)	27 (11.9)	29 (12.2)	31 (12.1)	
Yes		858 (87.9)	225 (87.9)	200 (88.1)	208 (87.8)	225 (87.9)	
Alcohol drinking							0.442
No		293 (30.0)	34 (30.1)	28 (30.0)	29 (30.0)	27 (30.1)	
Yes		683 (70.0)	179 (69.9)	159 (70.0)	166 (70.0)	179 (69.9)	
Death							<0.001
Yes		724 (74.2)	189 (73.8)	200 (88.1)	183 (77.2)	152 (59.4)	
No		252 (25.8)	67 (26.2)	27 (11.9)	54 (22.8)	104 (40.6)	
RT dose							<0.001
Median, Gy			70.00	0	70.00	63.00	
IQR			[35.03, 70.40]	0	[35.00, 70.20]	[0.00, 69.30]	
Follow-up duration							<0.001
Median, d		450.50	466.00	200.00	457.00	602.50	
IQR		[166.50, 783.50]	[208.25, 639.25]	[78.00, 572.00]	[178.00, 780.00]	[240.00, 1040.75]	

RT, radiotherapy; CCRT, concurrent chemoradiotherapy; CCI, Charlson Comorbidity Index; SD, standard deviation; IQR, interquartile range; AJCC, American Joint Committee on Cancer; TNM, tumor, node, and metastasis.

**Table 2:** Cox Proportional Hazards Regression Analysis of Death Risk among Elderly Patients (aged ≥70 years) with Locally Advanced Oral Cavity Squamous Cell Carcinoma.

	Univariate Analysis		Multivariate Analysis *	
Variables	HR (95% CI)	*p* Value of Type III Test	aHR (95% CI)	*p* Value of Type III Test
Treatment		<0.001		<0.001
CCRT (Ref.)	1.00		1.00	
No treatment	1.41 (1.15, 1.72)		1.60 (1.30, 1.97)	
RT alone	1.00 (0.82, 1.23)		1.06 (0.87, 1.31)	
Surgery	0.66 (0.53, 0.81)		0.69 (0.55, 0.85)	
Sex		0.005		0.092
Female (Ref.)	1.00		1.00	
Male	1.35 (1.1, 1.67)		1.21 (0.97, 1.52)	
Age		<0.001		<0.001
70 to 80 (Ref.)	1.00		1.00	
≥81	1.41 (1.17, 1.70)		1.57 (1.30, 1.90)	
AJCC clinical stage		<0.001		0.002
III (Ref.)	1.00		1.00	
IV	1.82 (1.5, 2.21)		1.24 (0.93, 1.65)	
AJCC clinical T stage		<0.001		<0.001
T1 (Ref.)	1.00		1.00	
T2	1.45 (0.97, 2.16)		1.45 (0.97, 2.16)	
T3	1.54 (1.03, 2.29)		1.79 (1.19, 2.69)	
T4	2.04 (1.41, 2.96)		2.16 (1.46, 3.21)	
AJCC clinical N stage		<0.001		<0.001
N0 (Ref.)	1.00		1.00	
N1	1.04 (0.83, 1.3)		1.27 (1, 1.62)	
N2	1.55 (1.28, 1.88)		1.60 (1.29, 1.99)	
N3	2.1 (1.43, 3.08)		2.27 (1.5, 3.44)	
Academic center		0.049		0.057
Nonacademic center	1.00		1.00	
Academic center	0.63 (0.40, 1.00)		0.64 (0.4, 1.01)	
CCI score		0.085		0.081
0 (Ref.)	1.00		1.00	
1	1.72 (0.89, 2.47)		1.58 (0.99, 2.28)	
2	0.98 (0.78, 1.23)		0.99 (0.78, 1.25)	
3	1.58 (1.10, 2.27)		1.18 (0.82, 1.71)	
4+	1.36 (1.02, 1.82)		1.28 (0.95, 1.74)	
Cigarette smoking		0.065		0.051
No (Ref.)	1.00		1.00	
Yes	1.37 (0.98, 1.92)		1.14 (0.99, 1.62)	
Betel nut use		0.034		0.05
No (Ref.)	1.00		1.00	
Yes	1.62 (1.04, 2.52)		1.55 (1.00, 2.43)	
Alcohol drinking		0.039		0.072
No (Ref.)	1.00		1.00	
Yes	1.31 (1.01, 1.7)		1.28 (0.98, 1.68)	

* All aforementioned variables were used in multivariate analysis. CCRT, concurrent chemoradiotherapy; CCI, Charlson Comorbidity Index; CI, confidence interval; aHR, adjusted hazard ratio; RT, radiotherapy; AJCC, American Joint Committee on Cancer; Ref., reference group.

**Table 3:** Cox Proportional Hazards Model for Mortality Risk in Patients 70 to 80 Years Old with Locally Advanced Oral Cavity Squamous Cell Carcinoma.

	Univariate Analysis		Multivariate Analysis *	
Variables	HR (95% CI)	*p* Value	aHR (95% CI)	*p* Value
		of Type III Test		of Type III Test
Treatment		<0.001		<0.001
CCRT (Ref.)	1.00		1.00	
No treatment	1.30 (1.03, 1.63)		1.52 (1.21, 1.91)	
RT alone	0.95 (0.76, 1.19)		1.02 (0.81, 1.28)	
Surgery	0.61 (0.48, 0.77)		0.66 (0.52, 0.83)	
Sex		0.004		0.301
Female (Ref.)	1.00		1.00	
Male	1.43 (1.12, 1.83)		1.15 (0.88, 1.49)	
AJCC clinical stage		<0.001		0.049
III (Ref.)	1.00		1.00	
IV	1.94 (1.55, 2.42)		1.39 (1, 1.94)	
AJCC clinical T stage		<0.001		0.003
T1 (Ref.)	1.00		1.00	
T2	1.29 (0.84, 1.99)		1.30 (0.84, 2.02)	
T3	1.44 (0.94, 2.21)		1.71 (1.1, 2.66)	
T4	1.89 (1.27, 2.82)		1.89 (1.24, 2.89)	
AJCC clinical N stage		<0.001		<0.001
N0 (Ref.)	1.00		1.00	
N1	1.10 (0.85, 1.43)		1.30 (0.99, 1.71)	
N2	1.67 (1.34, 2.07)		1.55 (1.21, 1.99)	
N3	2.19 (1.44, 3.34)		2.21 (1.39, 3.51)	
Academic center		0.132		0.096
Nonacademic center	1.00		1.00	
Academic center	0.70 (0.44, 1.11)		0.66 (0.41, 1.08)	
CCI score		0.223		0.569
0 (Ref.)	1.00		1.00	
1	1.63 (0.98, 2.49)		1.53 (0.99, 2.36)	
2	0.91 (0.70, 1.17)		0.91 (0.70, 1.18)	
3	1.60 (0.97, 2.40)		1.13 (0.75, 1.7)	
4+	1.30 (0.94, 1.8)		1.24 (0.88, 1.75)	
Cigarette smoking		0.219		0.119
No (Ref.)	1.00		1.00	
Yes	1.58 (0.98, 2.33)		1.30 (0.97, 1.94)	
Betel nut use		0.081		0.112
No (Ref.)	1		1.00	
Yes	1.66 (0.91, 3.22)		1.93 (0.86, 3.22)	
Alcohol drinking		0.131		0.106
No (Ref.)	1.00		1.00	
Yes	1.63 (0.91, 2.20)		1.56 (0.94, 2.15)	

* All aforementioned variables were used in multivariate analysis. CCRT, concurrent chemoradiotherapy; CCI, Charlson Comorbidity Index; CI, confidence interval; aHR, adjusted hazard ratio; RT, radiotherapy; AJCC, American Joint Committee on Cancer; Ref., reference group.

**Table 4:** Cox Proportional Hazards Model for Mortality Risk in the Oldest Patients Aged ≥ 81 years with Locally Advanced Oral Cavity Squamous Cell Carcinoma.

	Univariate Analysis		Multivariate Analysis *	
Variables	HR (95% CI)	*p* Value	aHR (95% CI)	*p* Value
		of Type III Test		of Type III Test
Treatment		0.115		0.277
CCRT (Ref.)	1.00		1.00	
No treatment	2.00 (1.26, 3.18)		1.83 (1.11, 2.40)	
RT alone	1.28 (0.79, 2.06)		1.12 (0.8, 1.26)	
Surgery	0.92 (0.56, 1.50)		0.90 (0.48, 1.13)	
Sex		0.347		0.207
Female (Ref.)	1.00		1.00	
Male	1.22 (0.8, 1.86)		1.37 (0.84, 2.23)	
AJCC clinical stage		0.038		0.856
III (Ref.)	1.00		1.00	
IV	1.53 (1.02, 2.29)		0.94 (0.49, 1.81)	
AJCC clinical T stage		0.154		0.211
T1 (Ref.)	1.00		1.00	
T2	2.22 (0.77, 6.34)	0.138	1.96 (0.62, 6.14)	
T3	2.17 (0.75, 6.32)	0.154	2.13 (0.65, 6.98)	
T4	3.07 (1.11, 8.54)	0.031	3.23(1.01, 7.38)	
AJCC clinical N stage		0.124		0.139
N0 (Ref.)	1.00		1.00	
N1	0.78 (0.49, 1.25)		1.23 (0.68, 2.22)	
N2	1.30 (0.85, 1.97)		1.71 (0.93, 2.86)	
N3	2.19 (0.85, 5.64)		2.60 (0.96, 6.03)	
Academic center		0.202		0.493
Nonacademic center	1.00		1.00	
Academic center	0.28 (0.40, 1.99)		0.48 (0.60, 3.9)	
CCI score		0.233		0.241
0 (Ref.)	1.00		1.00	
1	1.90 (0.91, 3.94)		1.75 (0.77, 3.96)	
2	1.91 (0.96, 3.31)		1.43 (0.79, 2.58)	
3	1.50 (0.66, 3.44)		1.47 (0.59, 3.68)	
4+	1.84 (0.96, 3.53)		1.49 (0.71, 3.14)	
Cigarette smoking		0.426		0.836
No (Ref.)	1.00		1.00	
Yes	1.35 (0.65, 2.79)		0.90 (0.35, 2.34)	
Betel nut use		0.941		0.706
No (Ref.)	1.00		1.00	
Yes	1.04 (0.37, 2.92)		0.80 (0.26, 2.49)	
Alcohol drinking		0.484		0.383
No (Ref.)	1.00		1.00	
Yes	0.8 (0.43, 1.49)		0.74 (0.37, 1.46)	

* All the aforementioned variables were used in multivariate analysis. CCRT, concurrent chemoradiotherapy; CCI, Charlson Comorbidity Index; CI, confidence interval; aHR, adjusted hazard ratio; RT, radiotherapy; AJCC, American Joint Committee on Cancer; Ref., reference group.

## Supplementary Materials

The following are available online at https://www.mdpi.com/2072-6694/12/2/258/s1, Figure S1: Cox proportional hazards model curves for overall survival of elderly patients (≥70 years) with locally advanced oral cavity squamous cell carcinoma receiving surgery and concurrent chemoradiotherapy, as obtained using the inverse probability of treatment weighting in an adjusted Kaplan–Meier method (adjusted for age; sex; Charlson Comorbidity Index; medical center; betel nut use; cigarette smoking; alcohol consumption; American Joint Committee on Cancer clinical stage; and tumor, node, and metastasis stage), Figure S2: Cox proportional hazards model curves for overall survival of elderly patients (≥70 years) with locally advanced oral cavity squamous cell carcinoma receiving radiotherapy alone and concurrent chemoradiotherapy, as obtained using the inverse probability of treatment weighting in an adjusted Kaplan–Meier method (adjusted for age; sex; Charlson Comorbidity Index; medical center; betel nut use; cigarette smoking; alcohol consumption; American Joint Committee on Cancer clinical stage; and tumor, node, and metastasis stage), Figure S3: Cox proportional hazards model curves for overall survival of elderly patients (≥70 years) with locally advanced oral cavity squamous cell carcinoma receiving nontreatment and concurrent chemoradiotherapy, as obtained using the inverse probability of treatment weighting in an adjusted Kaplan–Meier method (adjusted for age; sex; Charlson Comorbidity Index; medical center; betel nut use; cigarette smoking; alcohol consumption; American Joint Committee on Cancer clinical stage; and tumor, node, and metastasis stage).

## Abbreviations

aHR	Adjusted hazard ratio
AJCC	American Joint Committee on Cancer
CCI	Charlson comorbidity index
CCRT	Concurrent chemoradiotherapy
CI	Confidence interval
HNSCC	Head and neck squamous cell carcinoma
HR	Hazard ratio
ICD-9-CM	International Classification of Diseases, Ninth Revision, Clinical Modification
LA-OCSCC	Locally advanced oral cavity squamous cell carcinoma
NCCN	National Comprehensive Cancer Network
OCSCC	Oral cavity squamous cell carcinoma
OS	Overall survival; PS, propensity score
PSM	Propensity score–matched
RCT	Randomized clinical trial
RT	Radiotherapy
TCRD	Taiwan Cancer Registry database
TNM	Tumor, node, metastasis
