# Supplementary material for: Outcomes for Elderly Patients Aged 70 to 80 Years or Older with Locally Advanced Oral Cavity Squamous Cell Carcinoma: A Propensity Score–Matched, Nationwide, Oldest Old Patient–Based Cohort Study
Source: Cancers (Basel). 2020 Jan 21;12(2):258. doi: 10.3390/cancers12020258 (PMC7072345; doi:10.3390/cancers12020258)
Supplement: Supplementary file 1 [file cancers-12-00258-s001.pdf]

Supplementary Materials

Outcomes for Elderly Patients Aged 70 to 80 Years or Older with Locally Advanced Oral Cavity Squamous Cell Carcinoma: A Propensity Score–Matched, Nationwide, Oldest Old Patient–Based Cohort Study

Ben-Chang Shia, Lei Qin, Kuan-Chou Lin, Chih-Yuan Fang, Lo-Lin Tsai, Yi-Wei Kao and Szu-Yuan Wu

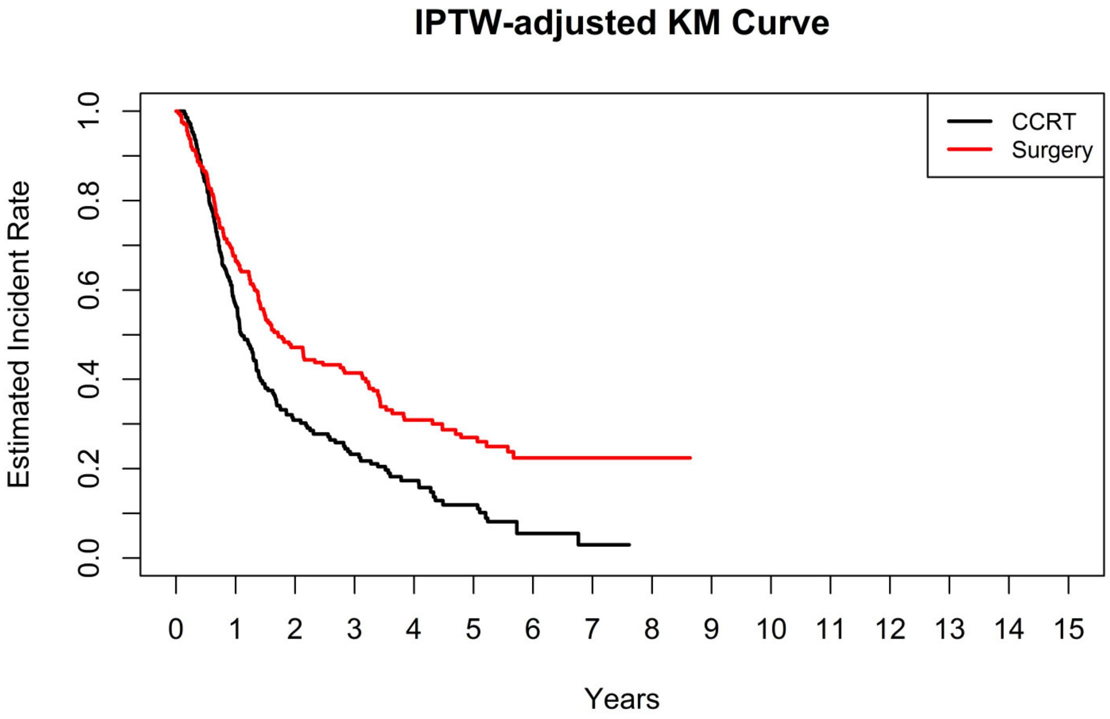

Figure S1. Cox proportional hazards model curves for overall survival of elderly patients (≥70 years) with locally advanced oral cavity squamous cell carcinoma receiving surgery and concurrent chemoradiotherapy, as obtained using the inverse probability of treatment weighting in an adjusted Kaplan–Meier method (adjusted for age; sex; Charlson Comorbidity Index; medical center; betel nut use; cigarette smoking; alcohol consumption; American Joint Committee on Cancer clinical stage; and tumor, node, and metastasis stage).

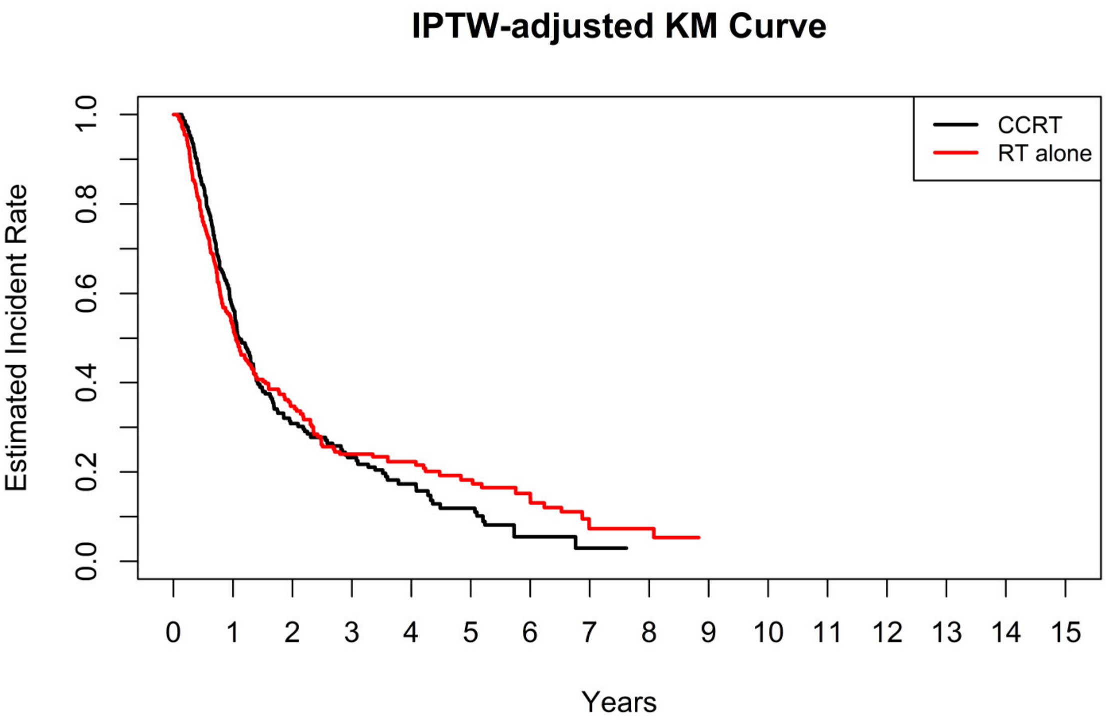

Figure S2. Cox proportional hazards model curves for overall survival of elderly patients (≥70 years) with locally advanced oral cavity squamous cell carcinoma receiving radiotherapy alone and concurrent chemoradiotherapy, as obtained using the inverse probability of treatment weighting in an adjusted Kaplan–Meier method (adjusted for age; sex; Charlson Comorbidity Index; medical center; betel nut use; cigarette smoking; alcohol consumption; American Joint Committee on Cancer clinical stage; and tumor, node, and metastasis stage).

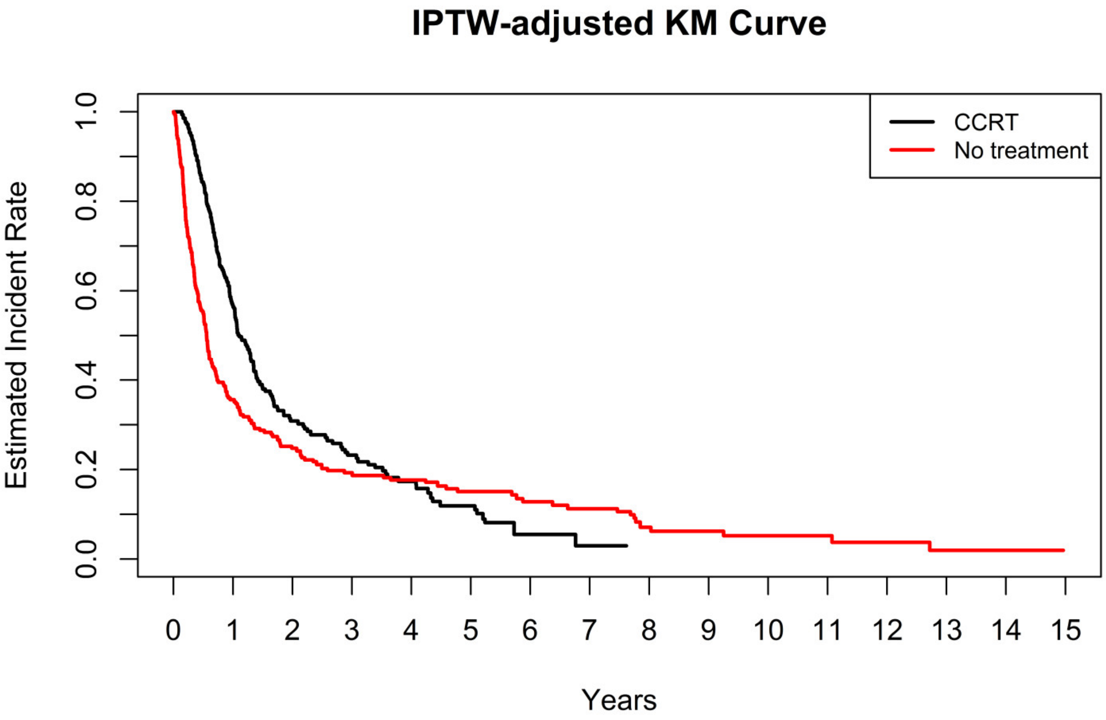

Figure S3. Cox proportional hazards model curves for overall survival of elderly patients (≥70 years) with locally advanced oral cavity squamous cell carcinoma receiving nontreatment and concurrent chemoradiotherapy, as obtained using the inverse probability of treatment weighting in an adjusted Kaplan–Meier method (adjusted for age; sex; Charlson Comorbidity Index; medical center; betel nut

use; cigarette smoking; alcohol consumption; American Joint Committee on Cancer clinical stage; and tumor, node, and metastasis stage).

© 2020 by the authors. Licensee MDPI, Basel, Switzerland. This article is an open access article distributed under the terms and conditions of the Creative Commons Attribution (CC BY) license (http://creativecommons.org/licenses/by/4.0/).
